# Fatal Gastrointestinal Disorders Due to COVID-19: A Case Series

**DOI:** 10.7759/cureus.40286

**Published:** 2023-06-12

**Authors:** Junya Hagiwara, Naofumi Bunya, Keisuke Harada, Hiroshi Nakase, Eichi Narimatsu

**Affiliations:** 1 Intensive Care Unit, Sapporo Medical University, Sapporo, JPN; 2 Gastroenterology and Hepatology, Sapporo Medical University, Sapporo, JPN

**Keywords:** multiple organ dysfunction, acute graft vs host disease, gastrointestinal disorder, venovenous extracorporeal membrane oxygenation, covid-19

## Abstract

Patients with coronavirus disease 2019 (COVID-19) primarily develop respiratory symptoms, which sometimes can be serious. Respiratory failure is the most common cause of death from COVID-19. This disease also causes gastrointestinal (GI) symptoms. However, there are limited reports that severe GI disorders caused by COVID-19 can be fatal. Herein, we report three cases of fatal GI disorders caused by COVID-19. All patients developed severe pneumonia requiring mechanical ventilation and venovenous extracorporeal membrane oxygenation (V-V ECMO) support. The respiratory status improved, and the patients could be weaned off support. However, severe watery diarrhea (over a few liters per day) developed after the onset of respiratory failure. The CT, endoscopic, and pathological findings were similar to those observed in GI graft-versus-host disease (GI-GVHD). Despite various efforts, the GI disorders did not improve, and all patients died of multiple organ failures associated with sepsis due to intestinal mucosal disruption. COVID-19 can cause fatal GI disorders and may have similar characteristics to GI-GVHD. Further investigation will contribute to a comprehensive understanding of fatal GI disorders due to COVID-19.

## Introduction

Coronavirus disease 2019 (COVID-19) is caused by severe acute respiratory syndrome coronavirus 2 (SARS-CoV-2). Since its first case was reported in December 2019 in Wuhan, China, over 400 million people have been infected and nearly 6 million people have died worldwide [1]. In Japan, approximately 3.8 million people have been infected and 20,000 people have died as of February 2022 [2].

Fever and respiratory symptoms are common in COVID-19 [3]. SARS-CoV-2 infection can cause acute respiratory failure, with patients requiring mechanical ventilation and/or venovenous extracorporeal membrane oxygenation (V-V ECMO). In severe cases, it can lead to death [4-6]. Furthermore, gastrointestinal (GI) symptoms, such as anorexia, nausea, vomiting, abdominal pain, and diarrhea, have been reported in 16-50% of the cases [7,8]. However, there are limited reports on fatally severe GI disorders secondary to COVID-19; here, we report three cases of these disorders.

## Case presentation

Patient 1

A 62-year-old man tested positive for SARS-CoV-2 and was asymptomatic. On Day 2, he developed fever and fatigue and was admitted to the hospital for quarantine. He was initiated on ciclesonide inhalation, favipiravir, and tocilizumab. However, on Day 7, he required oxygen supplementation due to worsening respiratory distress; on Day 10, the patient's SpO2 deteriorated to 85% despite receiving 10 L/min of oxygen therapy via a reservoir mask. Therefore, on Day 11, invasive mechanical ventilation was initiated (Table 1); he was placed on V-V ECMO the same day and then transferred to our hospital. However, his respiratory status improved, and he was weaned off of V-V ECMO on Day 17. He developed watery diarrhea on Day 28, which reached a maximum amount of approximately 10 L/day (Table 2). Biopsy specimens of the colon and rectum obtained during an endoscopic examination for intractable diarrhea on Day 40 revealed the presence of apoptotic bodies, which is characteristic of GI graft-versus-host disease (GI-GVHD). Furthermore, the cytomegalovirus (CMV) antigenemia test was positive on Day 71, and ganciclovir treatment was initiated. However, the diarrhea did not improve; thus, we suspected that an immunological mechanism was involved. Accordingly, we administered methylprednisolone (1000 mg for three days) and infliximab (450 mg/day; 5 mg/kg). We even performed a plasma exchange, but the GI disorder could not be controlled. The patient died on Day 142 owing to sepsis of intestinal origin and associated multiple organ failure.

**Table 1:** Baseline characteristics of cases * Vaccination had not yet been introduced.

	Patient 1	Patient 2	Patient 3
Age (years)	62	68	71
Gender (M/F)	M	M	M
BMI (kg/m^2^)	26	33	22.8
Comorbidities	Hypertension, diabetes, asthma	Hypertension, diabetes	Prostate cancer
Treatment	favipiravir, remdesivir, ciclesonide	favipiravir, ciclesonide	
Vaccination	none *	none	none
Initial symptom	fever	fever	fever, fatigue
Murray/RESP/Preserve score	3/3/2	3/1/4	3.5/3/4
Duration of ECMO support (days)	18	41	8
Outcome	death	death	death
Hospital stay (days)	142	87	60

**Table 2:** Characteristics of gastrointestinal disorders of each case ** IHC; immunohistochemistry

		Patient 1	Patient 2	Patient 3
GI symptom onset from developing COVID-19 (days) (days from intubation)		28 (17)	29 (17)	16 (13)
Initial symptom (maximum amount of diarrhea per day)		Watery diarrhea (10 L/day)	Watery diarrhea (3 L/day)	Watery diarrhea (7 L/day)
GI hemorrhage		+	+	+
Extent of GI disorders		Stomach – rectum especially far from descending portion of duodenum, mucosal sloughing was prominent	Stomach – rectum especially far from descending portion of duodenum, mucosal sloughing was prominent	Stomach – rectum especially far from descending portion of duodenum, mucosal sloughing was prominent
CT findings		Diffuse wall thickening of small bowel	Diffuse wall thickening of small bowel	Diffuse wall thickening of small bowel
Endoscopic findings				
	Upper GI	Stomach: mucosal edema Duodenum: extensive mucosal sloughing	Stomach: mucosal edema Duodenum: multiple ulcers	Stomach: mucosal edema Duodenum: multiple ulcers and extensive mucosal sloughing
	Lower GI	Colon: mucosal erythema and edema	Colon: extensive erosion and mucosal sloughing Rectum: multiple ulcers	Colon: mucosal erythema and edema
Pathological findings (days from onset)		Apoptotic bodies, and mucosal sloughing from ascending, descending colon, and rectum (24)	Mucosal sloughing from rectum (38)	Apoptotic bodies and mucosal sloughing from rectum (19)
CMV enteritis		Antigenemia (+), IgG(+), IgM(-), PCR(-), IHC**(-)	Antigenemia (-), IgG(+), IgM(-), PCR(-), IHC(-)	Antigenemia (-), IgG(+), IgM(-), PCR(+), IHC(-)
Enterocolitis due to Clostridioides difficile		Glutamate dehydrogenase (-), toxinA/B (-)	Glutamate dehydrogenase (-), toxinA/B (-)	Glutamate dehydrogenase (-), toxinA/B (-)
Non-occlusive mesenteric ischemia		-	-	-
Treatment		High-dose methylprednisolone, IVIG, infliximab, plasma exchange, ganciclovir treatment	Plasma exchange, ganciclovir treatment	High-dose methylprednisolone, IVIG, infliximab, ganciclovir treatment

Patient 2

A 68-year-old man developed a fever and had a positive polymerase chain reaction (PCR) result for SARS-CoV-2 on Day 5 after onset. On Day 7, the patient was admitted to the hospital and was initiated on oxygen therapy due to a decrease in SpO2. However, due to a rapid deterioration in the patient's respiratory status, mechanical ventilation was initiated the next day. He was started on ciclesonide inhalation and favipiravir; however, his respiratory failure worsened, and he was transferred to our hospital on Day 12 for V-V ECMO. On Day 29, he developed watery diarrhea of approximately 2 L/day. We suspected a *Clostridioides difficile* infection or CMV enteritis; however, tests for both were negative.

**Figure 1:**
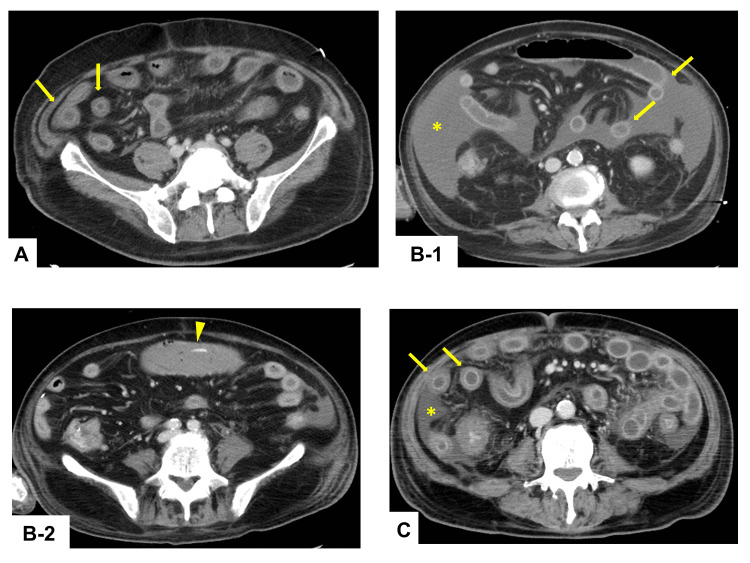
CT findings of each case (A) Patient 1: Day 33. (B-1) Patient 2: Day 51. (B-2) Patient 2: Day 56. (C) Patient 3: Day 32. Axial contrast-enhanced CT images show diffuse bowel wall thickening throughout the small bowel (arrows), along with ascites (asterisks), and small bowel hemorrhage (arrowhead).

**Figure 2:**
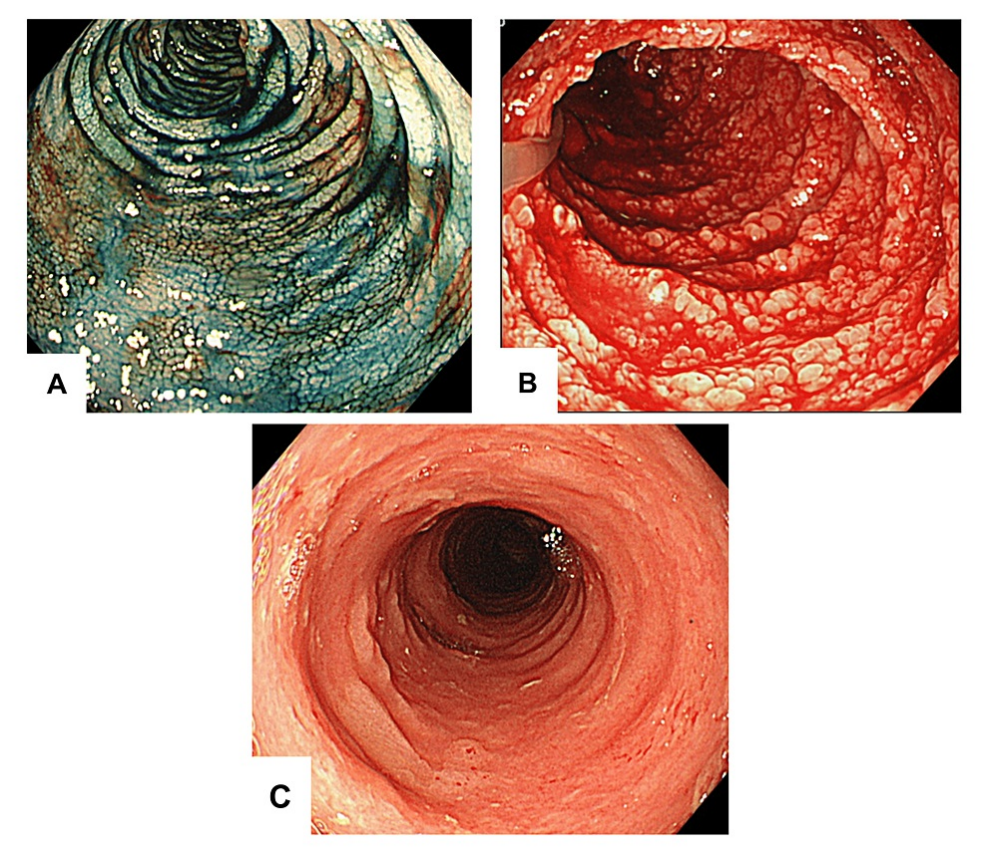
Endoscopic findings of each case (A) Patient 1: colonoscopy with indigo carmine dye spraying. Mucosal edema in the ascending colon has an “orange peel appearance.” (B) Patient 2: extensive mucosal edema and erosions,  and diffuse bleeding in the duodenum. (C) Patient 3: mucosal sloughing in the colon.

Contrast-enhanced CT revealed edematous wall thickening of the entire bowel, especially the small intestine (Figure 1). There was no evidence of intestinal ischemia, such as non-occlusive mesenteric ischemia. A conference was held with gastroenterologists to discuss the possibility of other GI diseases being associated with autoimmune diseases. Despite performing plasma exchange, diarrhea did not improve. Endoscopic findings revealed gastric mucosal edema, multiple duodenal ulcers, and diffuse mucosal sloughing from the terminal ileum to the rectum (Figure 2). Conversely, his respiratory condition improved gradually, and he was weaned off V-V ECMO on Day 42.

On Day 56, he developed hemorrhagic shock, and contrast-enhanced CT revealed a small bowel hemorrhage (Figure 1). We performed transcatheter arterial embolization and temporarily controlled the bleeding. However, the bleeding recurred the following day, and emergency surgery and partial small bowel resection were performed. Watery diarrhea persisted, and an immunological mechanism was suspected as the cause; therefore, plasma exchange was performed. However, diarrhea did not improve, and the patient experienced repeated sepsis due to disruption of the intestinal mucosa; he died of multiple organ failure on Day 87.

Patient 3

**Figure 3:**
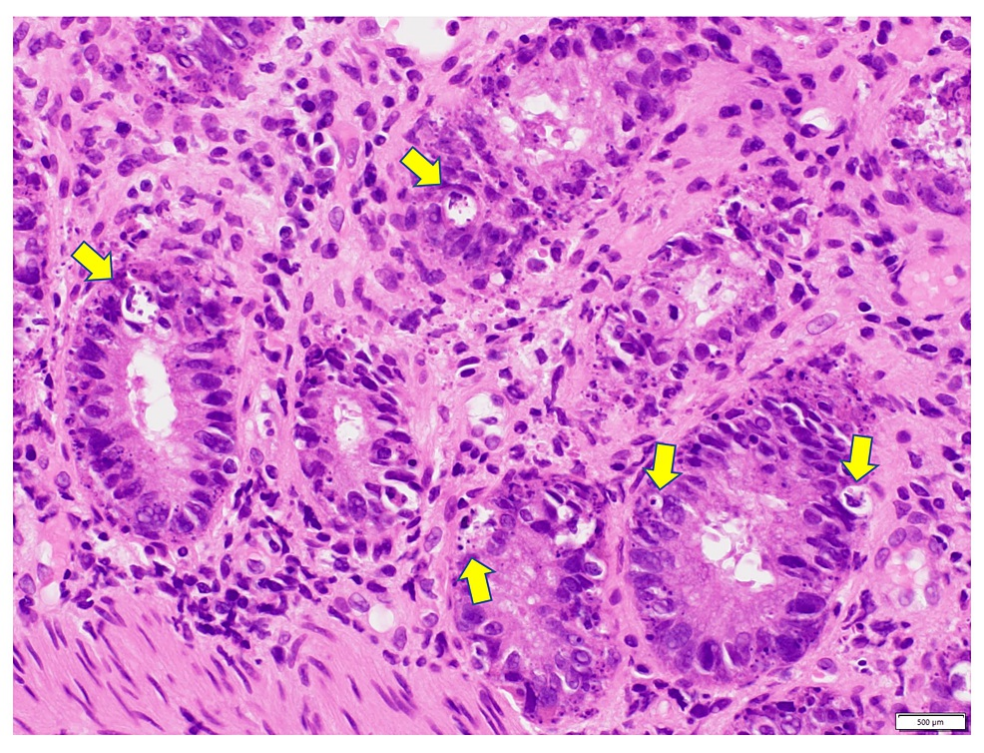
Pathological findings of Patient 3 The rectal biopsy specimen shows apoptotic bodies in the crypts (arrows; original magnification: hematoxylin-eosin, ×400).

A 71-year-old man developed fever, fatigue, shortness of breath the following day, and hypoxemia. He was transferred to the emergency department, and invasive mechanical ventilation was initiated. The PCR assay was positive for SARS-CoV-2 on Day 2, and the patient was transferred to our hospital for V-V ECMO. On Day 9, the patient was decannulated because his respiratory status had improved. However, he developed watery diarrhea of approximately 4 L/day on Day 14. We started him on intravenous immunoglobulin therapy (IVIG; 20 g/day; 400 mg/kg) and high-dose methylprednisolone (1000 mg/day); however, diarrhea remained uncontrolled. Histopathological examination of biopsy specimens of the colon and rectum, obtained during an endoscopic examination for intractable diarrhea on Day 20, confirmed the presence of apoptotic bodies (Figure 3). The colon specimen was positive for CMV-PCR on Day 32; thus, treatment with ganciclovir was initiated. However, despite initiating treatment with infliximab, the GI disorder did not improve, and the patient died of multiple organ failure on Day 60.

## Discussion

Our cases indicate that COVID-19 can cause fatal GI disorders. The characteristics of these disorders may be similar to those of GI-GVHD. GVHD is caused by donor lymphocytes that recognize host histocompatibility antigens as foreign bodies and attack them immunologically. The main target organs are the skin, liver, and GI tract. The clinical symptoms of GI-GVHD generally include watery diarrhea, nausea, vomiting, and anorexia. The pathogenesis of GVHD is thought to be due to direct damage to the mucosa of the GI tract by donor cytotoxic T cells and due to tissue damage by cytokines [9]. Regarding the imaging findings for GI-GVHD, contrast-enhanced CT has revealed abnormal mucosal enhancement of patchy or diffusely thickened bowel segments, especially in the small intestine, in approximately 75% of the cases [10]. The characteristic endoscopic findings of GI-GVHD include mucosal edema, erythema, and fragility [11]. The endoscopic findings of the stomach and duodenum can range from normal-to-mild edema or erythema to dramatic mucosal sloughing [12]. The ileum, cecum, and ascending colon are common sites in the lower GI tract, and villi atrophy, erythema, edema, erosions, and broad ulcer-like lesions are known to occur [9]. An “orange peel appearance” and “tortoise shell-like” mucosa are characteristic findings in the colon [12]. Histopathological features include apoptosis of the epithelial cells and lymphocytic infiltration [9], which are prominent at the base of the crypt where the intestinal epithelium proliferates. In mild or early cases, the presence of apoptotic bodies is the only clue for a GI-GVHD diagnosis [13]. However, in severe cases, crypt abscesses, crypt disappearance, and mucosal sloughing may be observed [9,11,14].

In all of our cases, diffuse bowel wall thickening was observed in the small intestine on a CT scan. Upper GI endoscopy revealed mucosal edema in the stomach, extensive mucosal sloughing, and multiple duodenal ulcers. Lower GI endoscopic findings revealed extensive mucosal sloughing in the small intestine, including the terminal ileum; extensive mucosal edema in the colon; and mucosal sloughing and multiple ulcers in the rectum. The CT and endoscopic findings were similar to those for GI-GVHD. Histologically, in Patient 1, there was ulceration and villous atrophy in the terminal ileum and apoptotic bodies in the ascending and descending colon and rectum. In Patient 2, we found mucosal sloughing in the rectum. In Patient 3, the presence of apoptotic bodies was confirmed in the rectum.

All these histological findings were similar to those of GI-GVHD. Galimberti et al. reported that COVID-19 is characterized by a cytokine storm and hyperactivation of the immune response, similar to that observed with acute GVHD, resulting in damage to various organs [15]. Murphy reported that in both acute GVHD and COVID-19, critical epithelial stem cell populations are preferentially targeted in the early phases of the disease [16]. Furthermore, both conditions are mediated, in part, by cytokines, with a cytokine storm playing an important role in further driving and amplifying the disease pathogenesis. Yamakawa et al. reported the case of a patient focusing on extensive mucosal sloughing of the entire intestine and the elevated expression levels of IL-6 in the small intestine and colon [17]. In their case report, the mention of similarity to GI-GVHD was partial. In the present report, the additional two cases with similar lesion extent, endoscopic findings, and pathological findings allowed us to clarify the characteristics of this fatal gastrointestinal disorder.

Ozawa et al. reported the case of a patient with severe COVID-19 who required ECMO; the endoscopic and histopathological findings of GI disorders were similar to those of GVHD [18]. They also reported that GI disorders improved after administering steroids and infliximab. These findings are consistent with our clinical findings. Our patients’ conditions did not improve despite the administration of IVIG, high-dose methylprednisolone, and infliximab. This may have been due to the delay in starting these treatments. Therefore, early diagnosis and treatment are critical.

Recently, a multisystem inflammatory syndrome in children and adults (MIS-C/MIS-A) was proposed to describe a hyperinflammatory state that can occur four to six weeks after primary SARS-CoV-2 infection in children and adults [19]. Our patients developed GI disorders that were delayed from the onset of respiratory failure due to COVID-19; this implies that they were not caused by SARS-CoV-2 infection directly, but by some post-infection immune dysregulation, and may be included in MIS-A. Patients 1 and 2 represented cases of early COVID-19 outbreaks in Japan when there were no reports of fatal GI disorders associated with COVID-19. We have previously reported on the possible involvement of MIS-A in terms of re-exacerbation of the inflammatory response and multiorgan disorders [20].

## Conclusions

COVID-19 can cause fatal GI disorders and may share similarities with GI-GVHD. CT, endoscopic, and pathological findings are useful for recognizing fatal GI disorders secondary to COVID-19. Further investigations will contribute to a comprehensive understanding of these disorders.
